# Hydrophilicity, Viscoelastic, and Physicochemical Properties Variations in Dental Bone Grafting Substitutes

**DOI:** 10.3390/ma11020215

**Published:** 2018-01-30

**Authors:** Branko Trajkovski, Matthias Jaunich, Wolf-Dieter Müller, Florian Beuer, Gregory-George Zafiropoulos, Alireza Houshmand

**Affiliations:** 1 Wound Healing and Oral Diagnostic Research Group, College of Dental Medicine, University of Sharjah, Sharjah 27272, UAE; 2 Botiss Biomaterials GmbH, Hauptstr. 28, 15806 Zossen, Germany; 3 Bundesanstalt für Materialforschung und-Prüfung (BAM), Unter den Eichen 44-46, 12203 Berlin, Germany; matthias.jaunich@bam.de; 4 Charité Centrum Zahn-, Mund-und Kieferheilkunde, Charité-Universitätsmedizin Berlin, Aßmannshauser Straße 4-6, 14197 Berlin, Germany; wolf-dieter.mueller@charite.de (W.-D.M.); florian.beuer@charite.de (F.B.); houshmand@mkg-westend.de (A.H.); 5 Preventive and Restorative Dentistry Department, College of Dental Medicine, University of Sharjah, Sharjah 27272, UAE; GGZafi@gmx.de

**Keywords:** biomaterials, bone grafting, bone substitutes, hydrophilicity, mechanical analysis

## Abstract

The indication-oriented Dental Bone Graft Substitutes (DBGS) selection, the correct bone defects classification, and appropriate treatment planning are very crucial for obtaining successful clinical results. However, hydrophilic, viscoelastic, and physicochemical properties’ influence on the DBGS regenerative potential has poorly been studied. For that reason, we investigated the dimensional changes and molecular mobility by Dynamic Mechanical Analysis (DMA) of xenograft (cerabone^®^), synthetic (maxresorb^®^), and allograft (maxgraft^®^, Puros^®^) blocks in a wet and dry state. While no significant differences could be seen in dry state, cerabone^®^ and maxresorb^®^ blocks showed a slight height decrease in wet state, whereas both maxgraft^®^ and Puros^®^ had an almost identical height increase. In addition, cerabone^®^ and maxresorb^®^ blocks remained highly rigid and their damping behaviour was not influenced by the water. On the other hand, both maxgraft^®^ and Puros^®^ had a strong increase in their molecular mobility with different damping behaviour profiles during the wet state. A high-speed microscopical imaging system was used to analyze the hydrophilicity in several naturally derived (cerabone^®^, Bio-Oss^®^, NuOss^®^, SIC^®^ nature graft) and synthetic DBGS granules (maxresorb^®^, BoneCeramic^®^, NanoBone^®^, Ceros^®^). The highest level of hydrophilicity was detected in cerabone^®^ and maxresorb^®^, while Bio-Oss^®^ and BoneCeramic^®^ had the lowest level of hydrophilicity among both naturally derived and synthetic DBGS groups. Deviations among the DBGS were also addressed via physicochemical differences recorded by Micro Computed Tomography, Scanning Electron Microscopy, Fourier Transform Infrared Spectroscopy, X-ray powder Diffractometry, and Thermogravimetric Analysis. Such DBGS variations could influence the volume stability at the grafting site, handling as well as the speed of vascularization and bone regeneration. Therefore, this study initiates a new insight into the DBGS differences and their importance for successful clinical results.

## 1. Introduction

The use of bone grafting materials has significantly increased since the first principles of grafting were established in the 1900s [1]. In fact, about 2.2 million bone grafting procedures are carried out worldwide every year with estimated costs of about USD 2.5 billion [2]. A significant part of these procedures involves the use of autogenous bone being associated with limited sources and an increased risk of donor site morbidity [3]. For that reason, various bone graft substitutes have been developed as alternative to autogenous bone. For example, the European market for Dental Bone Graft Substitutes (DBGS) in 2001 was estimated at USD 20.5 million, which included Germany, France, Italy, Spain, and UK only [4]. The number of placed dental implants due to the aging European population, as well as new market developments, continuously increased over the years. Therefore, the European market for DBGS and related products exceeded USD 200 million in 2013 and continues to grow even further [5]. On the other hand, the global market for dental membranes and bone graft substitutes is expected to rise from US $419 million in 2015 to US $922.6 million by 2024 [6]. 

The DBGS are essentially bone void fillers that allow bone in-growth and have to fulfil basic requirements such as biocompatibility, osteoinduction or osteoconduction, porosity, stability under stress, easy handling, resorbable or not resorbable, plasticity, safety & sterility, and long-term stable integration and success [2,3]. Different classes of DBGS (autogenic, allogenic, xenogenic, and synthetic), their advantages and disadvantages, as well as examples with various products and certain indications, have already been described in detail [7]. The use of DBGS requires the correct classification of the bone defects and the recommendation of an appropriate treatment plan [8]. This is of high importance, especially when clinical success relies on a highly technical, sensitive procedure [9]. 

The lack of indication-oriented DBGS selection, as well as the presentation of the basic requirements of DBGS as advantages, can be a reason for negative results, which is hardly being addressed during implant failures [10]. For example, the hydrophilic SLA (Sand blasted Long grit Acid-etched) implant surfaces demonstrated significantly a higher mean percentage of bone-implant contact when compared to the controls [11]. In such a case, the increased degree of hydrophilicity was correlated to increased implant stability and an enhanced level of osseointegration [12]. Consequently, hydrophilicity became one of the most addressed DBGS properties [13]. Still, there is a lack of data addressing the DBGS hydrophilicity degree and its influence on their regenerative potential. 

The viscoelastic material properties and mechanical loading affect the cell/tissue response towards the scaffold remodeling that can impact the regeneration process [14]. Especially, the DBGS production methods can affect the porosity, surface area, and mechanical strength that can lead to differences in new bone formation and graft bioresorption [9]. However, clinical studies mostly focus on limited DBGS properties rather than showing the influence of physico-chemical differences due to origin and processing methods. For example, Demineralized Bone Matrix (DBM), in combination with hyaluronic acid, inorganic bovine bone, and synthetic beta-tricalcium phosphate ß-TCP formulations, were compared in order to analyze the beneficial effect and clinical effectiveness of the DBM [15]. The new bone formation and incorporated bone grafting materials analysis after 8 months gave less satisfactory results when ß-TCP was present. However, it is of great interest if both DBM and DBM/bovine bone groups would have long-term comparable results due to differences in the viscoelastic and DBGS resorption properties. 

The origin and processing methods of DBGS can cause differences in the hydrophilicity, viscoelastic, and physicochemical properties that can influence their handling, regenerative potential, and clinical outcome. For that reason, we performed Dynamic Mechanical Analysis (DMA) of xenograft, synthetic, and allograft blocks in a wet and dry state. Here, we observed the dimensional changes and molecular mobility of DBGS blocks during DMA. In addition, we compared the hydrophilicity behavior of xenograft and synthetic DBGS granules. The hydrophilicity analysis by high speed microscopy imaging included fresh animal blood behavior upon contact with the granules. Finally, a physico-chemical analysis of xenograft, synthetic, and allograft DBGS was performed in order to correlate the hydrophilicity and viscoelastic differences.

## 2. Materials and Methods

### 2.1. Blocks

Cerabone^®^ cancellous blocks 20 mm × 20 mm × 10 mm (sintered bovine), maxresorb^®^ blocks 20 × 20 × 10 mm (synthetic bi-phasic 60% hydroxyapatite (HA) and 40% beta-tricalcium phosphate (β-TCP)), and maxgraft^®^ cancellous blocks 20 mm × 10 mm× 10 mm (allograft bone from living donors, treated without acetone [16]) were provided by botiss biomaterials GmbH, Zossen, Germany. For mechanical testing comparison, Puros^®^ cancellous blocks 20 mm × 20 mm × 10 mm (allograft bone from cadaver donors, treated with acetone [17], Zimmer Dental GmbH, Munich, Germany) were also included in the study. When required, the blocks’ size was adjusted by a 10 mm diamond saw (botiss biomaterials GmbH).

### 2.2. Granules

The tested inorganic-bovine xenograft granules were cerabone^®^ 0.5–1 mm (sintered, botiss biomaterials GmbH, Zossen, Germany), Bio-Oss^®^ 0.25–1 mm (non-sintered, Geistlich Pharma AG, Wolhusen, Switzerland), and NuOss^®^ 0.25–1 mm (non-sintered, Ace Surgical Supply Inc., Brockton, MA, USA). For comparison, another naturally derived granule from phycogenic origin SIC^®^ nature graft 0.3–1 mm (SIC Invent AG, Basel, Switzerland) were also included. The selected synthetic grafting granules were maxresorb^®^ 0.5–1 mm (bi-phasic 60% HA and 40% β-TCP, botiss biomaterials GmbH, Zossen, Germany), Straumann^®^ BoneCeramic 0.5–1 mm (bi-phasic 60% HA and 40% β-TCP, Institute Straumann AG, Basel, Switzerland), NanoBone^®^ 0.6 mm (nanocrystalline HA embedded in silica gel matrix, Artoss GmbH, Rostock, Germany), and Ceros^®^ 0.7–1.4 mm (pure β-TCP, Mathys Ltd., Bettlach, Switzerland). The analyzed allograft granules were maxgraft^®^ 0.5–2 mm (allograft bone from living donors, treated without acetone [16], botiss biomaterials GmbH, Zossen, Germany). All granules were used as received. 

### 2.3. Dynamic Mechanical Analysis

To analyze the dimensional changes and molecular mobility of DBGS blocks in wet and dry state, the blocks were precisely sectioned to a 10 mm × 10 mm × 5 mm, and then five specimens of each group were mechanically tested by Dynamic Mechanical Analysis (DMA). DMA is a scientific mechanical testing technique that can be used for determination of the viscoelastic material properties under dynamic load conditions [18]. The measuring principle relies on oscillating stress being applied to the specimen as its displacement is then measured and recorded. Consequently, the phase shift between stress and strain is detected and used to calculate the damping behaviour of the material (tan δ). The measurements were performed by DMA 242 C Dynamic mechanical analyser (Netzsch-Gerätebau GmbH, Selb, Germany) as penetration specimen-holder equipped with a cylindrical tip of 3 mm diameter was used to study the time dependent behaviour and specimen height under presence or absence of water.

**Figure 1:**
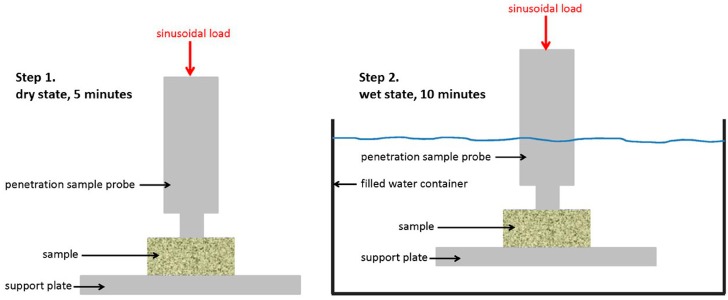
Schematic description of the method for Dynamic Mechanical Analysis (DMA).

For that reason, each specimen was initially calibrated until good contact with the probe was achieved. The specimens were then tested for five minutes (dry state) in order to determine the corresponding stiffness, specimen dimension changes due to applied load (creep), and damping behaviour. The testing dynamic force was 5 N with amplitude of 5 µm and frequency of 1 Hz. After five minutes, the specimens were submerged in water at room temperature (wet state) and the same measurements were applied for another ten minutes, which was considered to be a sufficient amount of wetting time (Figure 1).

### 2.4. Hydrophilicity Analysis by High Speed Microscopy Imaging

**Figure 2:**
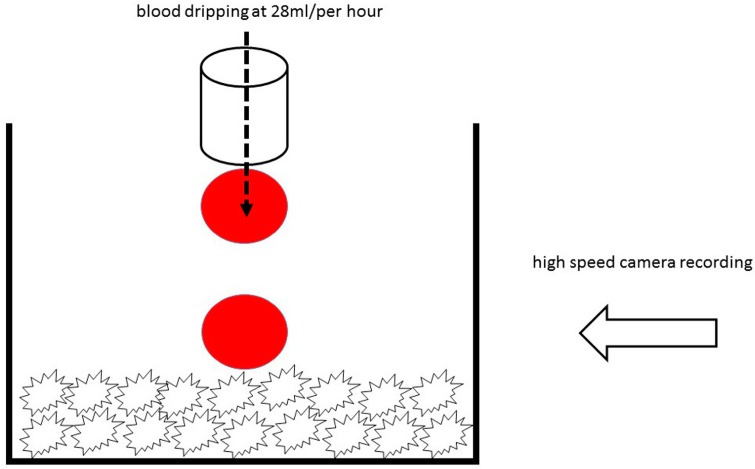
Schematic description of the method for hydrophilicity analysis.

Since DMA analysis could not be used to measure differences in hydrophilicity of the ceramic materials, the xenogenic and synthetic granules were placed in a homemade glass container, and their behavior was recorded by a high speed microscopy imaging under the angle of 90 degrees. Fresh porcine blood without anticoagulant and at room temperature was dripped over the granules by a 28 mL/per hour. Then slow motion videos were recorded at 125 frames per second with VW-6000E high speed microscopical imaging system (Keyence Deutschland GmbH, Neu-Isenburg, Germany). Consequently, six snapshots of each specimen were extracted, and the moment of first blood drop almost touching the granules was taken as the starting point (Figure 2). The brightness and contrast were later adjusted in order to better visualize the blood drop behavior. 

### 2.5. Physico-Chemical Analysis

The macroscopic geometry structure of the DBGS blocks was obtained by a Micro Computed Tomography-µCT (70 kVp, 114 mA; vivaCT 40, Scanco Medical, Brüttisellen, Switzerland). 

In order to analyze the surface roughness and particle size distribution by a Scanning Electron Microscopy (SEM), the DBGS granules were gently placed over the substrate followed by gold sputtering, which increases the conductivity of the particles and reduces the charging effects. 

Fourier Transform Infrared Spectroscopy (FTIR) was used in order to compare differences in chemical bonds of the DBGS. The IR measurements were carried out on kBr pellet with a FT-IR spectrometer. The spectra were measured in a wavelength range of 400–4000 cm^−1^ with a resolution of 2 cm^−2^. For each measurement 100 scans were summed up in total. 

The X-ray powder Diffractometry (XRD) was carried in order to gain information about the mineral phases and crystallinity of the materials. Therefore, the DBGS granules were grinded into powder and then were fixed on a carrier. Literature data from the data bank of the International Centre for Diffraction Data (ICDD) was used for peaks mapping.

The thermal stability of DBGS granules due to presence of phases other than mineral was analyzed by Thermogravimetry. The Thermogravimetric Analysis (TGA) was carried out in an air atmosphere starting from room temperature up to 1000 °C and with a heating rate of 5 °C/min.

## 3. Results

### 3.1. Dynamic Mechanical Analysis

**Figure 3:**
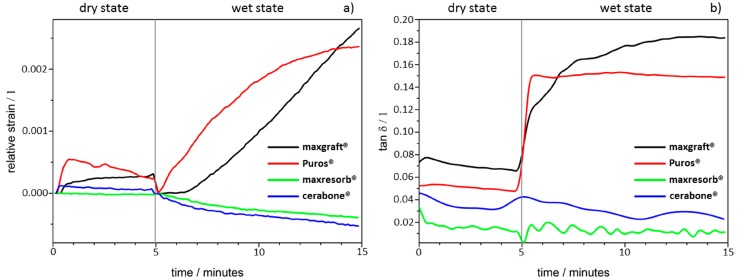
Dynamic Mechanical Analysis (DMA): (**a**) Dimensional changes; (**b**) molecular mobility changes.

The dimensional changes and molecular mobility of DBGS blocks depends on their origin, processing methods, and physico-chemical properties, which can affect their handling and regenerative potential. Therefore, different DBGS blocks were expected to have variations in their dimensional changes and molecular mobility in either wet or dry state (Figure 3).

During the initial-dry state testing for five minutes, only a slight height decrease with Puros^®^ was detected due to brittleness at the specimen contact surface and no differences were observed with the other groups (Figure 3a). However, the materials demonstrated different behaviour during the ten minutes of testing after water was applied. The cerabone^®^ and maxresorb^®^ blocks showed a slight height decrease (2–4 µm), whereas both maxgraft^®^ and Puros^®^ increased by ~10 µm with almost identical speed (Figure 3a). Apparently, the ceramic nature of cerabone^®^ and maxresorb^®^ leads to brittleness, which causes deformation at the specimen contact interface. On the other hand, the presence of organic content in the allografts caused water uptake and most probably is the reason for specimen swelling.

Furthermore, the cerabone^®^ and maxresorb^®^ blocks were highly rigid, and their damping behaviour was not influenced (Figure 3b). On contrary, both maxgraft^®^ and Puros^®^ had a strong increase in their molecular mobility but had different damping behaviour profiles. Puros^®^ almost immediately achieved equilibrium and there were no further changes in the tan δ during the tested period. In comparison, maxgraft^®^ had linear increase in tan δ and had higher values before achieving equilibrium. This indicates that maxgraft^®^ has more adaptable “rubber-like” structure in comparison to Puros^®^. This is probably due to higher hydrophilicity degree of maxgraft^®^ when compared to Puros^®^ that leads to higher water uptake capacity and therefore higher molecular mobility.

### 3.2. Hydrophilicity Analysis

**Figure 4:**
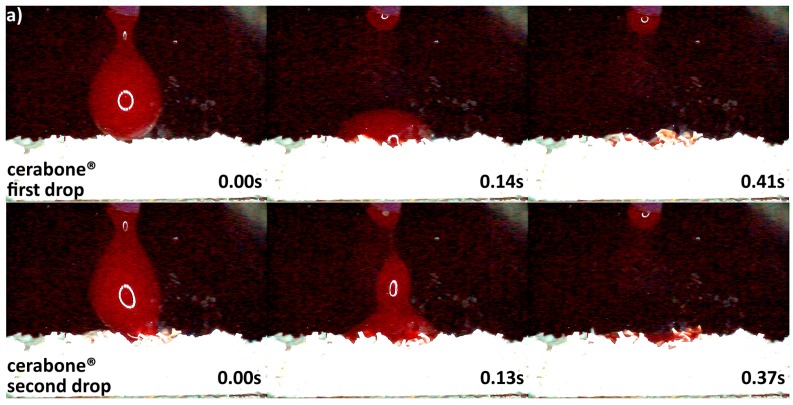

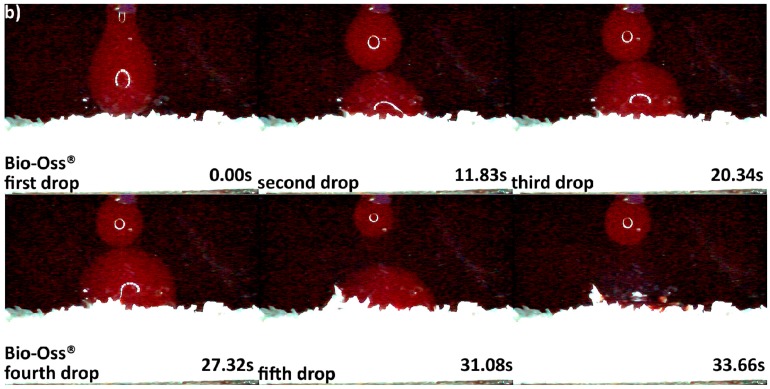
Hydrophilicity analysis: (**a**) cerabone^®^ 0.5–1 mm (fast blood uptake driven by capillary forces); (**b**) Bio-Oss^®^ 0.25–1 mm (blood adjustment over the surface followed by delayed leaking due to heavier drop formation).

**Figure 5:**
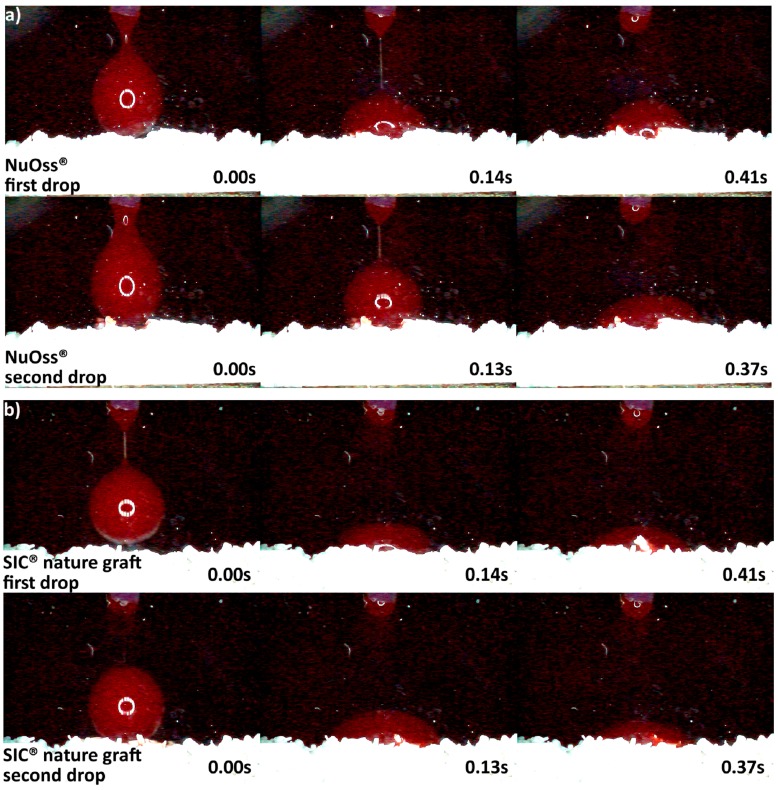
Hydrophilicity analysis: (**a**) NuOss^®^ 0.25–1 mm; (**b**) SIC^®^ nature graft 0.3–1 mm (blood adjustment over the surface of both materials followed by leaking).

The differences in origin, production process, and physico-chemical properties of the DBGS can lead to various hydrophilicity behaviors, which in turn could affect the handling and regenerative potential. We observed that cerabone^®^ was the only grafting material among the tested materials being able to “take-up” blood by capillary forces and that therefore had highest hydrophilicity degree (Figure 4a). More specifically, the first blood drop was completely “taken” by cerabone^®^ within only 0.41 s. The capillary forces were clearly seen with the second blood drop that was completely “taken” within 0.37 s upon contact with the granules. On the other hand, NuOss^®^ and SIC^®^ nature graft were less hydrophilic than cerabone^®^, as the first and second drops were “taken” only by half within 0.41 and 0.37 s, respectively (Figure 5). Most probably, that is a result of blood leaking between the granules rather than being “taken” by capillary forces as seen with cerabone^®^. On the contrary, Bio-Oss^®^ showed lowest hydrophilicity degree where the blood was “taken” after 33.66 s and only after accumulation of four drops into one bigger and heavier drop (Figure 4b). Apparently, the blood was only “leaking” between the Bio-Oss^®^ granules with lack of capillary forces.

**Figure 6:**
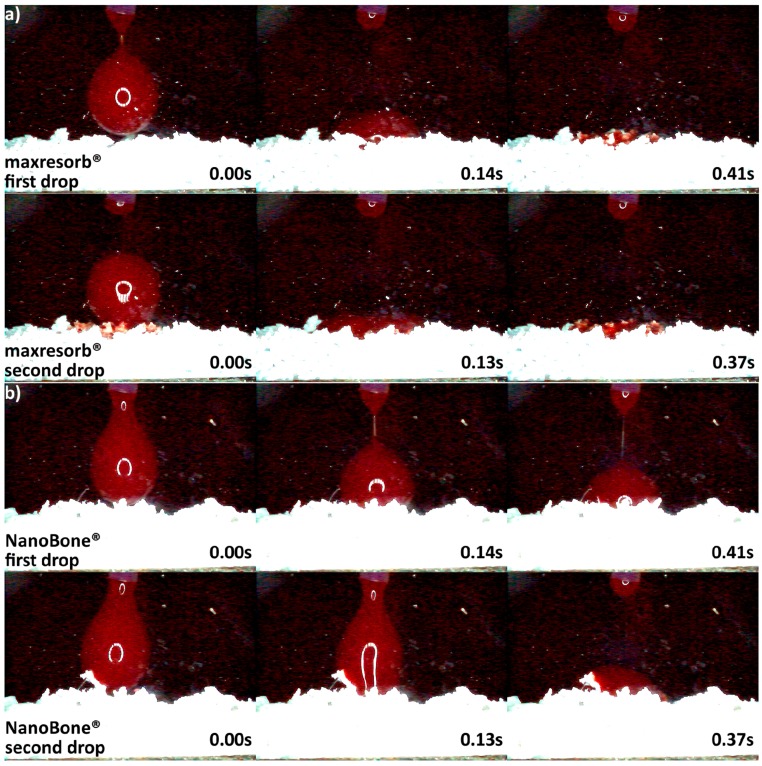
Hydrophilicity analysis: (**a**) maxresorb^®^ 0.5–1 mm (fast uptake of blood once in contact with the surface); (**b**) NanoBone^®^ 0.6 mm (blood adjustment over the surface followed by leaking).

**Figure 7:**
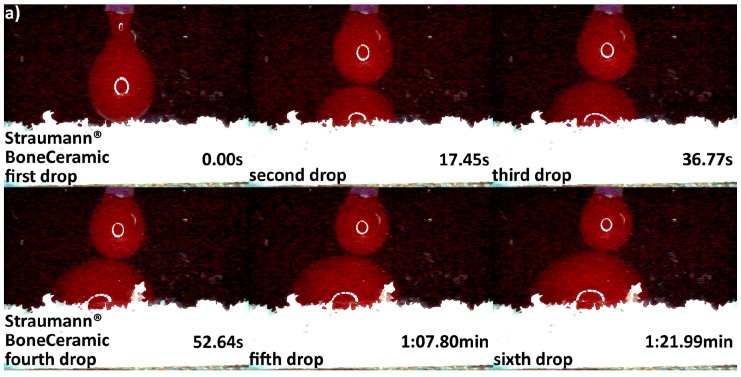

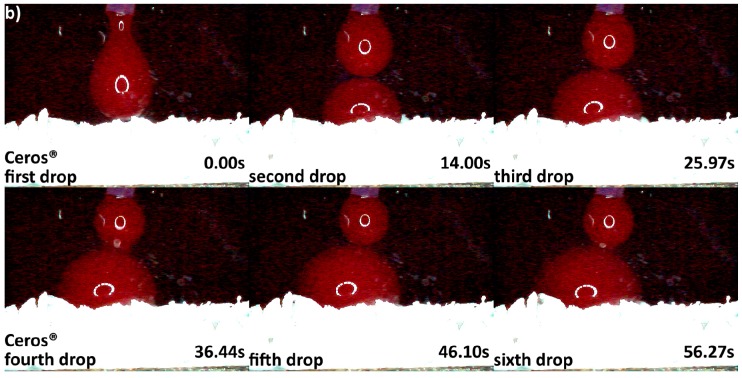
Hydrophilicity analysis: (**a**) Straumann^®^ BoneCeramic 0.5–1 mm (blood adjustment over the surface without any leaking during the tested period); (**b**) Ceros^®^ 0.7–1.4 mm (blood adjustment over the surface followed by delayed leaking after the sixth drop due to heavier drop formation).

Differences in hydrophilicity were also seen in the synthetic DBGS granules. Maxresorb^®^ showed highest hydrophilicity among the synthetic granules that was comparable to cerabone^®^. Even though no capillary forces were observed with maxresorb^®^, the first and second drops were completely “taken” upon contact and within 0.41 and 0.37 s, respectively (Figure 6a). On contrary, NanoBone^®^ was less hydrophilic because the blood drops adjusted over the surface followed by “leaking” between the granules, and the first and second drops were “taken” only about half within 0.41 and 0.37 s, respectively (Figure 6b). Furthermore, Ceros^®^ showed even lower hydrophilicity, in which at least six drops were required in order to accumulate over the surface that would later “leak” between the granules (Figure 7b). From all the groups, Straumann^®^ BoneCeramic showed lowest hydrophilicity, because the blood was not “taken” at all during the entire tested period (Figure 7a).

### 3.3. Micro Computed Tomography Analysis

**Figure 8:**
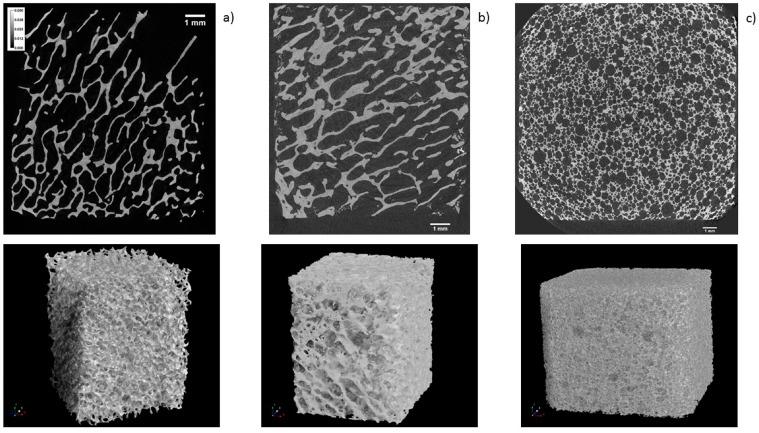
µCT analysis: (**a**) cerabone^®^ and (**b**) maxgraft^®^ have “labyrinth-like” cancellous bone structure; (**c**) maxresorb^®^ has “foam-like” structure with highly interconnected regular-circular pores. 1 mm ruler in every figure.

The regenerative potential and kinetics of new tissue deposition when using tissue engineering scaffolds depends on multiple factors, including their macroscopic geometry [19]. Therefore, µCT was used to compare the macroscopic geometry of xenograft, synthetic, and allograft DBGS blocks. The µCT analysis for both naturally derived DBGS cerabone^®^ (average porosity 40–65 vol %; 70% of the pores 300–1200 µm and 30% around 600–900 µm [20]) and maxgraft^®^ (allograft with preserved trabecular structure [21] if compared to intact human trabecular bone [22]) showed typical “labyrinth-like” cancellous bone structure (Figure 8a,b). On contrary, maxresorb^®^ (approximately 80% porosity, pore size 200–800 µm [23]) had “foam-like” structure with highly interconnected regular-circular pores (Figure 8c). 

### 3.4. Scanning Electron Microscopy Analysis

**Figure 9:**
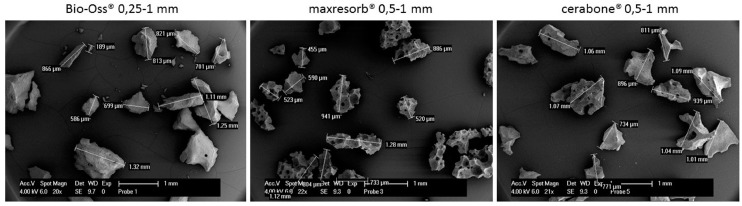
Particle distribution size analysis by Scanning Electron Microscopy SEM: Particles bigger than 1 mm were found in all samples and particles smaller than 0.25 mm were found in Bio-Oss^®^.

**Figure 10:**
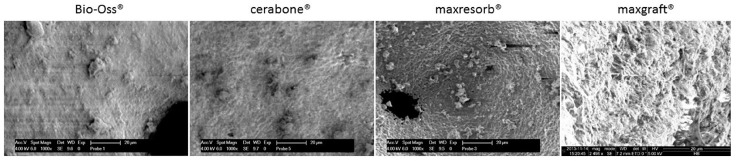
Particle surface analysis by Scanning Electron Microscopy SEM: cerabone^®^ shows rougher wave-like surface roughness when compared to Bio-Oss^®^; maxresorb^®^ has even rougher foam-like surface structure, and maxgraft^®^ has fiber-like surface structure due to presence of organic material. 20 µm ruler in every figure.

**Figure 11:**
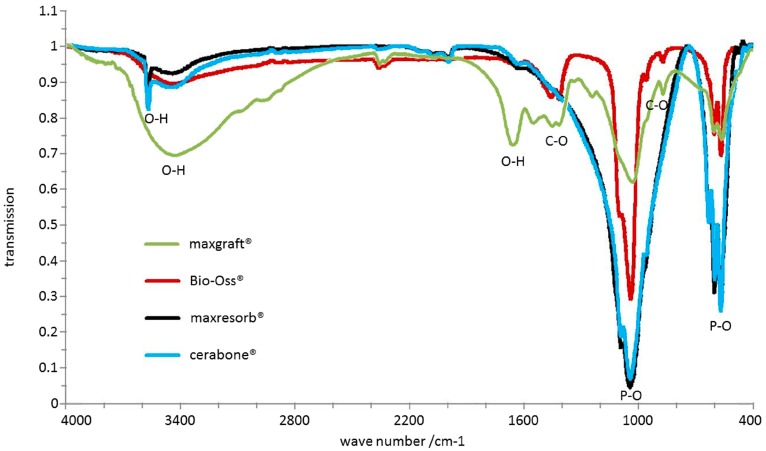
Chemical differences analyzed by FTIR: P-O bonds at 570–605 cm^−1^ and 970–1100 cm^−1^ as well as O-H bonds at ~3500 cm^−1^ and 1650 cm^−1^ were observed in all samples. cerabone^®^ and maxresorb^®^ showed additional hydroxyapatite O-H band around 3575 cm^−1^. 1460 cm^−1^, 1420 cm^−1^, and 880 cm^−1^ bands were observed for Bio-Oss^®^ and maxgraft^®^ due to presence of CO_3_^2−^ that were not present in cerabone^®^ and maxresorb^®^. maxgraft^®^ also had absorption bands at 1550 cm^−1^ and 1245 cm^−1^ corresponding to C-H and N-H bonds.

The particles distribution size and surface roughness of DBGS can be different from the specifications provided by the manufacturers, which can influence their inter-granulate porosity, osteoconductivity, and hydrophilicity. For that reason, bovine-derived particles cerabone^®^ and Bio-Oss^®^ were compared to synthetic granules maxresorb^®^ as they were dispersed and measured along their longest edge (Figure 9). While particles bigger than 1 mm were found in all specimens, Bio-Oss^®^ also contained many particles smaller than its given range of 0.25–1 mm (Figure 9). The irregular structure of cerabone^®^ particles was comparable to Bio-Oss^®^ (Figure 9) but had rougher wave-like surface (Figure 10). On the other hand, the maxresorb^®^ particles have “foam-like” structure (Figure 9) with even rougher “grain-like” surface (Figure 10). For comparison, the surface roughness of maxgraft^®^ particles had “fiber-like” structure (Figure 10), which is probably due to the presence of organic material (Figure 11 and Table 1). The particle distribution size of maxgraft^®^ was not measured due to fast allograft remodeling into new bone [16], in which the size of granules is not of such great relevance.

### 3.5. Chemical Structure, Mineral Phases, and Crystallinity Analysis

The origin and production process of the DBGS results leads to differences in chemical structure, mineral phases, and crystallinity that can affect the viscoelastic properties, hydrophilicity, and regenerative potential. Therefore, bovine-derived particles cerabone^®^ and Bio-Oss^®^ were compared to synthetic granules maxresorb^®^ and allograft granules maxgraft^®^.

FTIR is used to detect chemical differences between materials. All recorded spectra by FTIR showed several phosphate bands in the range of 570–605 cm^−1^ and 970–1100 cm^−1^ (Figure 11). Absorption bands around ~3500 cm^−1^ and 1650 cm^−1^ resulting from O-H-bonds due to water were also visible in all specimens. However, the recorded spectra of cerabone^®^ and maxresorb^®^ showed additional band around 3575 cm^−1^, which is caused by the O-H bonds of hydroxyapatite and was not observed in Bio-Oss^®^ and maxgraft^®^. On the contrary, additional bands at about 1460 cm^−1^, 1420 cm^−1^, and 880 cm^−1^ were observed in Bio-Oss^®^ and maxgraft^®^ due to presence of CO_3_^2−^ and were not detected in cerabone^®^ and maxresorb^®^. Due to presence of organic material like collagen, absorption bands at 1550 cm^−1^ and 1245 cm^−1^ corresponding to C-H and N-H bonds were seen only in maxgraft^®^.

**Figure 12:**
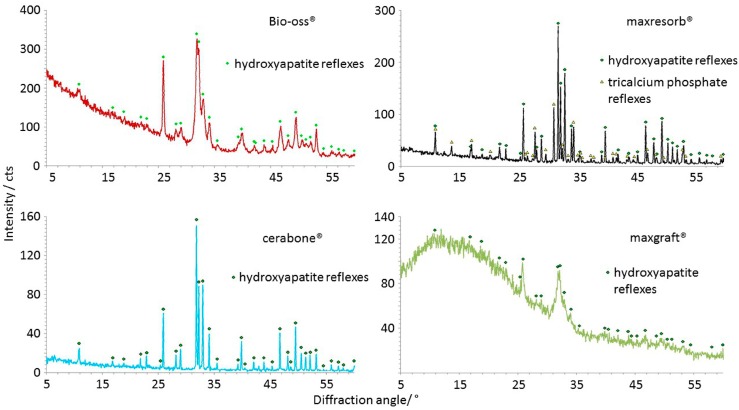
Crystallinity differences analyzed by XRD: Hydroxyapatite peaks were seen in all samples, and only maxresorb^®^ has additional reflexes due presence of β-tricalcium phosphate (Ca_3_(PO_4_)_2_). The narrow peaks and a low baseline indicate the high crystallinity of cerabone^®^ and maxresorb^®^. Bio-Oss^®^ shows broader peaks due lower crystallinity level. maxgraft^®^ also has broad peaks and a high baseline due low crystallinity and amorphous structure.

XRD is used to detect differences in mineral phases and crystallinity of materials. The XRD results showed presence of hydroxyapatite peaks in all diffractograms, and additional reflexes were detected in maxresorb^®^ due to the presence of β-tricalcium phosphate (Ca_3_(PO_4_)_2_) (Figure 12). While narrow peaks and a low baseline in both cerabone^®^ and maxresorb^®^ indicate high crystallinity, Bio-Oss^®^ showed broader peaks due to its lower crystallinity. Very broad peaks and a high baseline due to low crystallinity and amorphous structure were also seen in maxgraft^®^. Such broad peaks are probably caused by a carbonate integration within the crystal lattice of hydroxyapatite that was also confirmed by FTIR (Figure 11). 

Thermogravimetric analysis is used to detect impurities within materials caused by differences in origin and production process. The residual mass of maxresorb^®^ and cerabone^®^ after TGA was 98.6% and 99.5%, respectively, which indicates low water content and only a trace of carbon dioxide (Table 1). However, two separate phases following heating were seen in Bio-Oss^®^ with mass reduction of 3.3% and 4%, respectively. The first phase can be attributed to vaporization of the chemically bound water and the second phase to vaporization of carbon dioxide. Finally, maxgraft^®^ had residual mass of 61.48% that confirms the presence of organic material. 

## 4. Discussion

The aim of this study was to analyze variations in the hydrophilicity, viscoelastic, and physicochemical properties of few commercially available DBGS due to origin and production process. Such differences in DBGS can possibly influence the volume stability at the grafting site, the handling, and the speed of vascularization and bone regeneration. To analyze this, xenograft, synthetic, and allograft blocks were observed by DMA for dimensional changes and molecular mobility. Furthermore, the hydrophilicity behavior of xenograft and synthetic DBGS granules upon contact with fresh animal blood was compared by high speed microscopy imaging. Also, a physico-chemical analysis was performed in order to correlate the observed hydrophilicity and viscoelastic variations.

There is still lack of detailed indication-oriented knowledge of DBGS selection [8]. To specify, the properties such as biocompatibility, long-term stability, success, safety & sterility, easy handling, and osteoconductivity are very often being presented as advantages instead of as basic requirements that every DBGS should fulfill [7]. More specifically, there are four main classes of DBGS: autogenic, allogenic, xenogenic, and synthetic [2]. They are not different among each other only by origin, but also in terms of the diversity of processing methods such as extraction, sintering, chemical treatment, enzymatic digestion, low temperature treatment, synthesis, oxidative treatment, etc. The author of [7] can cause significant variations in composition, handling, resorption times, volume stability, and biologic activity, and can support various regeneration mechanisms. Personalized treatment plan with detailed interdisciplinary knowledge can allow indication-oriented and predictable patient treatment [24]. Therefore, this study was initiated to address the differences in hydrophilic, viscoelastic, and physicochemical properties of several DBGS (Table 2). 

The dimensional changes and molecular mobility of the DBGS depend on factors such as chemical composition and crystallinity that can possibly affect both handling and regenerative potential. For that purpose, xenograft, synthetic, and allograft DBGS blocks were tested by DMA in wet and dry state. Only Puros^®^ showed some signs of brittleness and no changes were seen in the other groups during the initial dry state testing (Figure 3). However, all groups showed changes in height and molecular mobility during the wet state testing. The purely ceramic materials cerabone^®^ and maxresorb^®^ had slight height decrease of 2–4 µm (Figure 3a), probably due to brittleness caused by their pure mineral and highly crystalline structure (Figure 8 and Figure 9). Their damping behaviour was not influenced (Figure 3b), which indicates high rigidity and stiffness. The lack of “amortizing effect” due to the absence of organic fraction causes brittleness and therefore questionable weight bearing and handling properties [1,25]. Consequently, such DBGS composition can crack upon shaping and fixation, but it provides better volume stability at the grafting site if compared to DBGS also having organic content [26]. 

The xenogenic (bovine) DBGS have naturally high crystalline (Figure 12) and rigid structure (Figure 3b) that causes low resorption time [27]. Such regenerative mechanism relies on penetration of newly formed bone into the DBGS porosity and its followed by integration [28]. Since the osteoclasts require very long time to dissolve the xenogenic (bovine) DBGS [29,30], the new bone formation within the porosity would be also delayed. However, variations in the production process of xenogenic (bovine) DBGS can cause morphological, chemical, crystallinity, and impurity differences (Figure 9, Figure 10, Figure 11 and Figure 12 and Table 1) that can affect the clinical results [29]. For instance, the sintering method causes increased crystalline size [31] that imparts better long-term volume stability of cerabone^®^ at the grafting site if compared to the low temperature [29] or the chemically treated bovine DBGS [32].

Furthermore, maxresorb^®^ has similar viscoelastic properties like cerabone^®^ (Figure 3), but the osteoclasts require much less time for its resorption [33]. This is probably caused by the synthetic hydroxyapatite structure and the presence of β-tricalcium phosphate Ca_3_(PO_4_)_2_ (Figure 12). The effect of very low biodegradation in hydroxyapatite as disadvantage is very well studied [3]. This limits the new bone formation that should replace the DBGS. Therefore, β-tricalcium phosphate has been added to hydroxyapatite to create biphasic material with higher bioresorption rate. Such regenerative mechanism relies on both new bone formation in the porosity and on actual remodelling of the DBGS into new bone [34]. The increase in DBGS porosity during remodelling is parallel mechanism to decreased architectural structure provided by the initial grafting [9]. In fact, the total porosity is expected to highly influence the total graft bioresorption by providing access to osteoclast and macrophages. Consequently, the new bone formation would be faster but with less long-term volume stability if compared to the bovine DBGS [35]. 

Variations in the production process for synthetic ceramic DBGS of one and same composition can lead to significant differences in the hydrophilicity (Figure 6 and Figure 7) that can possibly cause clinical effect. However, the synthetic ceramics still have certain advantages over the naturally derived DBGS because they can be prepared as foam-like circular porous structure (Figure 8c). According to the in vitro analysis, the pre-osteoblasts cells had different tissue deposition speed when cultivated in hydroxyapatite plates with square or cross-shaped sections [19]. Apparently, the initial tissue deposition within cross-shaped pores was twice faster than in the square-shaped pores. In both cases, the proliferating cells first tend to occupy corners followed by central circular formation within each individual pore. Therefore, a foam-like circular porous structure (Figure 8c) might possibly act as “proliferation time saving geometry” and be more favorable than irregular “labyrinth-like” cancellous structure (Figure 8a,b). Also, the porosity of synthetic ceramics can be adjusted in order to influence the volume of regenerated bone and quality of bone-implant integration [9].

On the other hand, the DMA analysis in wet state showed that maxgraft^®^ blocks increased in height ~10 µm (Figure 3a). This is probably due to water uptake and swelling caused by the organic content, low crystallinity, and amorphous structure (Figure 11 and Figure 12, Table 1). The Puros^®^ blocks showed almost identical height increase behaviour (Figure 3a), and there was a strong increase in the molecular mobility for the both groups, but with different damping profiles (Figure 3b). More specifically, maxgraft^®^ showed a linear increase in the tan δ and higher values when compared to Puros^®^ (Figure 3b). Such linear and higher water uptake capacity behaviour is most probably due to higher hydrophilicity degree. On contrary, Puros^®^ almost immediately reached equilibrium after wetting without any further changes in the tan δ (Figure 3b). This could be caused by surface water uptake only and delayed in depth absorption. Anyway, maxgraft^®^ seems to have more “rubber-like” structure if compared to Puros^®^, which indicates better adaptation to surroundings like tissues and implant surfaces. This is due to the variations in the allograft processing methods by the tissue banks [36]. For example, Puros^®^ has been completely dehydrated by acetone [17], unlike maxgraft^®^, which is non-acetone treated [16]. 

The presence of organic material (Figure 11 and Table 1), low crystallinity, and amorphous structure present in the allograft DBGS (Figure 12) leads to faster resorption time [37] and also provides less volume stability at the grafting site [38]. Such regenerative mechanism relies on parallel remodelling of the DBGS into newly formed bone [39]. As a result, the increase in porosity can lead to decreased volume and structural changes [38]. Still, the presence of low crystallinity and amorphous structure (Figure 12) imparts certain volume stability, while the organic content (Figure 11 and Table 1) contributes the faster new bone formation [37]. 

Variations in the production process of allograft DBGS [2] has effect on the viscoelastic material properties (Figure 3) that can also influence the scaffold’s regenerative potential [14]. For example, the Deminaralized Bone Matrix (DBM) is prepared from human donor bone by removal of its mineral phase and leaving behind collagen and non-collagenic proteins, e.g., growth factors [40]. Even though DBMs are widely used for dental bone regeneration, their use and efficiency remain controversial because of their “osteo-inductive index” variability that is not subject to strict control by the tissue banks [24]. In addition, multiple factors and their interaction rather than single features are likely to determine the DBM potency [41]. More specifically, the removal of the mineral phase has the purpose of exposing certain growth factors that can actually have misleading marketing purpose due to questionable therapeutic dosages and biologic activity. Finally, the mineralized allografts and their mineral “space maintaining” content still provide better volume stability at the grafting site if compared to DBM [38]. 

Variations in the origin and processing DBGS methods affect their hydrophilicity (Figure 3b and Figure 4, Figure 5, Figure 6 and Figure 7), which can influence their handling and regenerative potential. The DMA analysis showed hydrophilicity differences between the allografts, because they had distinct wet-state molecular mobility profiles, and such variations could not be observed for the purely ceramic groups (Figure 3b). For that reason, the hydrophilicity degree of xenograft and synthetic DGBS granules was recorded by a high speed camera with fresh blood under an angle of 90 degrees (Figure 4, Figure 5, Figure 6 and Figure 7).

The highest hydrophilicity was observed in cerabone^®^ in which capillary forces could be detected (Figure 4). This is most probably due to pure mineral ~100% hydroxyapatite composition (Figure 11), high crystallinity (Figure 12), very low impurities level (Table 1), and the 0.5–1 mm distribution size that creates greater inter-granulate porosity between the cerabone^®^ granules. The physicochemical properties of NuOss^®^ and SIC^®^ nature graft were not tested in this study; however, their lower hydrophilicity (Figure 5) can most probably be attributed to the presence of <0.5 mm granules that decrease the inter-granulate porosity and delay the blood “leaking due gravity“ process. In addition, Bio-Oss^®^ showed lowest hydrophilicity among the tested xenograft DBGS (Figure 4). The missing hydroxyapatite O-H bonds at 3575 cm^−1^ and the presence of CO_3_^2−^ (Figure 11), the lower crystallinity level (Figure 12), and impurities such as chemically bound water and carbon dioxide (Table 1) could be the reason for such behavior. Also, the presence of <0.5 mm granules in Bio-Oss^®^ (Figure 9) decreases its inter-granulate porosity, which delays even further the blood “leaking due gravity” process.

Furthermore, maxresorb^®^ showed highest hydrophilicity among the tested synthetic DBGS (Figure 6) and was similar to cerabone^®^. This might be due to additional ~3575 cm^−1^ band that both maxresorb^®^ and cerabone^®^ share, and it is caused by O-H bonds of the hydroxyapatite (Figure 11). However, no capillary forces were observed with maxresorb^®^, but the blood was taken up immediately upon contact with the granules (Figure 6) and is most probably due the presence of β-tricalcium phosphate (Ca_3_(PO_4_)_2_) (Figure 12). In addition, NanoBone^®^ had lower hydrophilicity, which can be attributed to presence of silica and lower inter-granulate porosity caused by the 0.6 mm granule distribution size (Figure 6). Moreover, ß-TCP Ceros^®^ showed even lower hydrophilicity, even though it has greater inter-granulate porosity provided by the 0.7–1.4 mm granules distribution size (Figure 7). Apparently the less crystalline ß-TCP mineral (Figure 12) is less hydrophilic than hydroxyapatite, which could be a reason for lack of capillary forces in maxresorb^®^ (Figure 6). Surprisingly, Straumann^®^ BoneCeramic showed completely different and rather hydrophobic behavior if compared to maxresorb^®^. In this case, the blood remained over the granules during the entire tested period and never “leaked” within the Straumann^®^ BoneCeramic (Figure 7). Further studies must be performed in order to determine what could cause this variation, even though both their 60%HA/40%β-TCP composition and 0.5–1 mm granule distribution size would suggest almost identical materials.

Apparently, both origin and processing methods of the DBGS influence the purity of the mineral phase [42], presence or absence of organic material [43], porosity [20], surface roughness [44], volume stability [29], and changes in crystallinity and crystals orientation [31]. Consequently, that would cause hydrophilicity variabilities and would influence the liquid uptake capacity and handling. Increased hydrophilicity not only enables more organized adsorption of blood and serum proteins, but it is of crucial importance for acquiring better implant stability and enhanced level of osseointegration [12]. However, there is not enough clinical research addressing the DBGS hydrophilicity variations and if it really has an effect on the speed of new blood vessels formation and bone regeneration.

## 5. Conclusions

Detailed analysis in the viscoelastic material properties, hydrophilicity, and physicochemical differences of several DBGS was performed. Such DBGS properties are strongly correlated with the origin and production process. As a result, we observed dimensional changes and molecular mobility of DBGS blocks in either a wet or dry state. Such differences could be of crucial importance for volume stability at the grafting site and handling. The observed variations in DBGS hydrophilicity could also have an effect on the speed of vascularization and bone regeneration. Therefore, more clinical studies are required in order to address the influence of the viscoelastic and hydrophilic DBGS properties at early and later healing stages. 

## Figures and Tables

**Table 1:** Residual masses following Thermogravimetric Analysis.

Specimen	Residual Mass in %
Bio-Oss^®^	92.64
maxresorb^®^	98.61
cerabone^®^	99.52
maxgraft^®^	61.48

**Table 2:** Summary of DGBS properties.

Properties	Xenograft	Synthetic	Allograft
Dimensional changes and molecular mobility (Figure 3)	High rigidity and stiff, brittleness due purely ceramic nature	High rigidity and stiff, brittleness due purely ceramic nature	Swelling due presence of organic material
Resorption rate	Low due highly crystalline natural HA structure	Medium due synthetic HA and ß-TCP structure	Fast due low crystallinity, amorphous structure and organic material presence
Volume stability at the grafting site	High due low resorption rate	Medium due to bi-phasic resorption rate	Low due fast resorption rate
Regenerative mechanism	Slow due penetration of newly formed bone and integration in the porosity	Medium due parallel new bone formation and remodelling	Fast new bone formation and remodelling due fast resorption rate and organic content
Hydrophilicity (Figure 3, Figure 4, Figure 5, Figure 6 and Figure 7)	Variations due mineral purity, crystallinity, particle distribution size	Variations due chemical structure, particle distribution size	Variations due acetone use during manufacturing
Macroscopic structure (Figure 8)	Labyrinth-like	Foam-like	Labyrinth-like
Particles structure and surface (Figure 9 and Figure 10)	Irregular structure and rough surface	Foam-like structure and grain-like surface	Irregular structure and fiber-like surface
Chemical structure (Figure 11)	P–O, O–H due water, additional hydroxyapatite O–H in cerabone^®^, additional CO_3_^2−^ in Bio-Oss^®^	P–O, O–H due water, additional hydroxyapatite O–H	P–O, O–H due water, CO_3_^2−^, C–H, N–H
Crystalline structure (Figure 12)	Hydroxyapatite, narrow peaks and a low baseline in cerabone^®^ due high crystallinity; broader peaks due lower crystallinity in Bio-Oss^®^	Hydroxyapatite, β-tricalcium phosphate, narrow peaks and a low baseline due high crystallinity	Hydroxyapatite, very broad peaks and a high baseline due low crystallinity and amorphous structure
Impurities (Table 1)	Low water content and only a traces of carbon dioxide in cerabone^®^; chemically bound water and carbon dioxide in Bio-Oss^®^	Low water content and only traces of carbon dioxide	Organic material
